# Beyond Measure: Human-Values-Based Care

**DOI:** 10.1007/s11606-025-10139-y

**Published:** 2026-01-21

**Authors:** Priyank Jain, David A. Hirsh

**Affiliations:** 1 Cambridge Health Alliance, 1493 Cambridge St, Cambridge, MA 02139 USA; 2 Harvard Medical School, 25 Shattuck Street, Boston, MA 02115 USA

**Keywords:** value-based care, professional virtues, patient-doctor relationship, Artificial intelligence, ethics, Phronesis

“The practice of medicine is an art, not a trade; a calling, not a business…”—Sir William Osler ^1^

The classical ethos of medicine as a *calling*, rooted in moral judgment and character, is confronting a modern medical industry focused on economic value and measurable outcomes. This strain may increase with the incorporation of artificial intelligence (AI) into the medical industry. In this viewpoint, we scrutinize how a strict focus on utility—the hallmark of value-based care and the foundation of AI integration—risks eroding physicians’ human capacity and compromises patient and physician wellbeing.

Historically, medicine has been understood as a profession, “devoted to the naturally given end of health and the assistance of the powers of self-healing”.^2^ In an era of simpler technology, the ministrations of a healer centered on compassion and guidance to the patient, and exercised virtues like honesty, kindness, and judgment.^3^ These were the relational and *humanistic* values that guided medical practice.

In the modern era, the medical industry was transformed by rapid scientific and technological advancements in diagnosis, treatment, and organizational science. In recent decades, value-based care has emerged as a dominant paradigm, incorporating “patient perceived value conferred by health care interventions for the resources expended”.^4^ Effectively, value-based care has *redefined* value as a mathematical derivation of outcomes and the inputs required to achieve them.^4^ This utilitarian approach to value in healthcare stems from a confluence of factors: rising healthcare costs demand demonstrable returns on investment; evidence-based medicine provides targets for population-level metrics; and regulatory requirements drive process measures for patient safety. This utilitarian system is enabled by abundant healthcare information technology which tracks patient and business metrics.

While these advances are well-intentioned, their collective emphasis on *industrial* value diverts the focus away from medicine’s humanistic and relational core.^5^ The richness of a patient’s experience is lost in Likert scales. When doctors apply careful clinical reasoning to align with their patient’s preferences and context, surrogate measures fail to capture the complexity. Process measures designed for benchmarking system performance are used for physician compensation and health system reimbursement. Cost and quality are managed through standardization and efficiency and cause friction with the patient’s individual needs. In short, value-based care has become a third party in the venerable patient-physician dyad, and has a *human* cost that is unquantifiable.

Consider a 40-year-old patient admitted for chest pain. She lacks a primary care physician (PCP) and has had multiple visits to the emergency department. After ruling out acute coronary syndrome, her pain resolves. She needs upper GI endoscopy and cardiac stress testing for diagnostic clarity, but these are deemed “inappropriate resource stewardship.” After a night in the hospital, she has no sure diagnosis and is discharged with advice to set up a PCP to coordinate further tests. She shares with the hospitalist, who is meeting her for the first time, “I’m scared. I don’t have a doctor. Could you help me understand why this happened? What does this all mean? What should I do?” The hospitalist has no satisfactory answer, and even if they did, caring care is not readily measured and not reimbursed. In this daily reality, efficiency pressures, compliance imperatives, and insurance constraints conflict with the physician’s professional responsibility to ensure diagnostic closure and patient assurance. System aims that excise care from caregiving drive physicians’ and patients’ moral distress.

System fragmentation also causes harm.^6^ The current health system boosts efficiency by segmenting hospitalists and primary care specialists. During her most vulnerable moments in the hospital and ED, this patient is seen by many different and unfamiliar doctors. The accountability for her overall experience diffuses when care is broken into episodic encounters. The hospitalist role is focused primarily on immediate tasks rather than longitudinal wellbeing, undermining the physician’s connection to deep, personal responsibility.^6^

Administrative burden, standardization, and fragmentation are reshaping the physician’s professional lives. Physicians experience distress when constrained from acting according to their moral judgment. Extrinsic rewards attached to measurable targets can crowd out the intrinsic joy that comes from problem-solving and compassionate care. In the industrial medical system, de-professionalized physicians lose resilience and risk burnout ^7^ (Fig. 1).

**Fig. 1:**
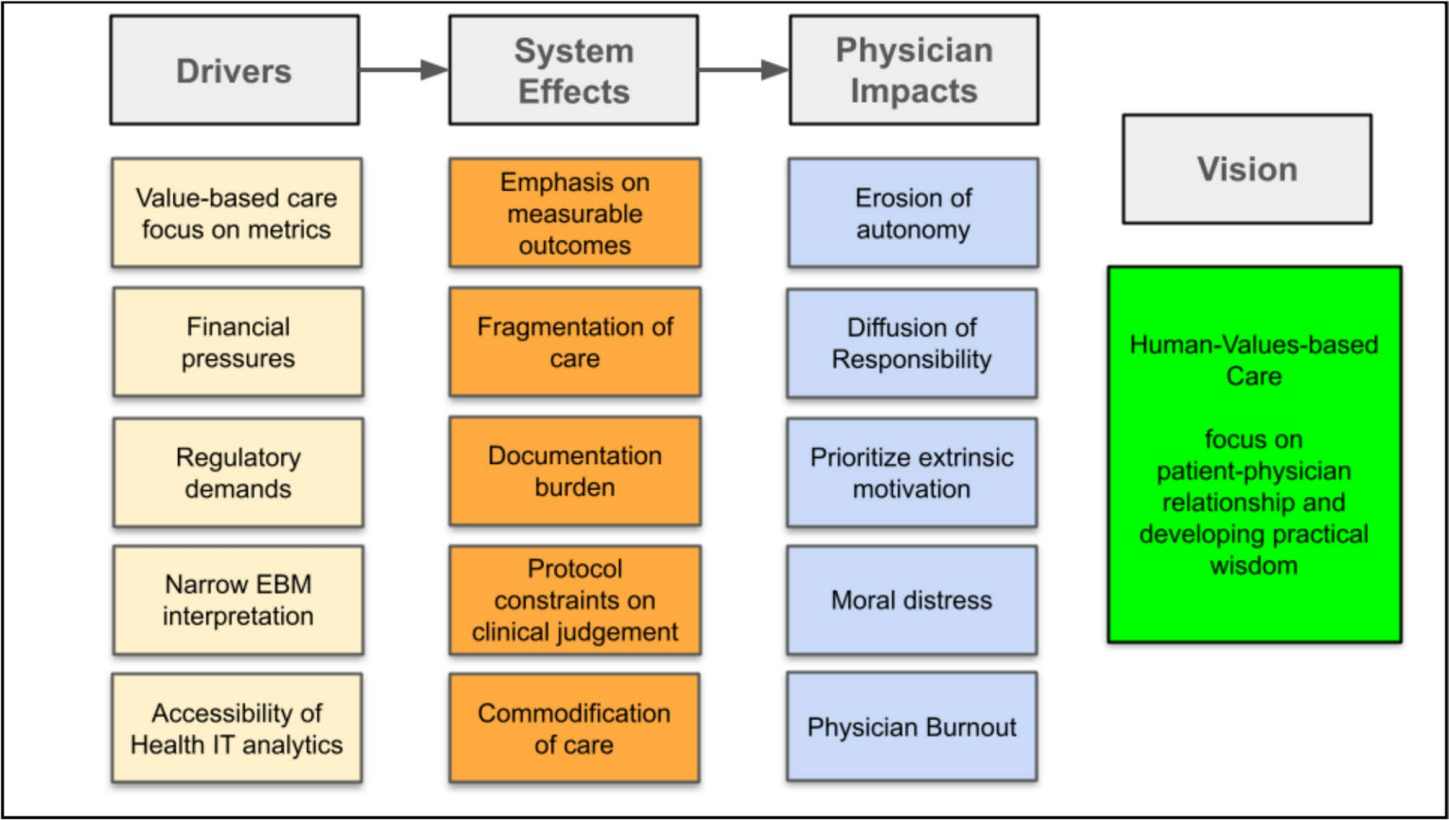
Impact of value-based care drivers on physicians and the health system

As the medical industry integrates AI into practice, the imposition of technology into physicians’ lives will increase. The physician role will be challenged as AI grows as a sovereign tool to expedite and enforce value-based care. AI can optimize measurement, streamline efficiency, and assume many of the cognitive and administrative tasks currently assigned to physicians. In this emerging context, we physicians should seize the opportunity to reinvest our freed-up cognitive space—and time—to provide the personal care patients seek and need. While AI replaces exhausting, time-consuming, and error-prone tasks, physicians may reclaim core elements of their professional roles: perception, human connection, compassion, and practical wisdom. While AI has a purpose, physicians need a *sense* of purpose.

Practical wisdom, the Hellenistic virtue called *Phronesis*, integrates purpose, judgment, and character.^3^ Human judgment equips the physician to address the nuanced social, spiritual, and individual needs that lie beyond the morally sterile domains of computation and metrics. Character provides the humanity and fortitude to prioritize the individual patient in the face of the compulsion and limitations of industrial utility. Medicine can emphasize practical wisdom to maintain the human art in caregiving, and the healing that flows from it. This healing restores the patient’s wholeness and dignity while ensuring the physician’s sense of purpose and professional flourishing. Our calling now is to safeguard this vision of human-values-based care.

## Data Availability

There are no data relevant to this viewpoint essay.
